# P-STAT3 Inhibition Activates Endoplasmic Reticulum Stress-Induced Splenocyte Apoptosis in Chronic Stress

**DOI:** 10.3389/fphys.2020.00680

**Published:** 2020-06-30

**Authors:** Manyu Song, Chaoran Wang, Haotian Yang, Yongping Chen, Xiujing Feng, Bei Li, Honggang Fan

**Affiliations:** College of Veterinary Medicine, Northeast Agricultural University, Harbin, China

**Keywords:** chronic stress, STAT3, apoptosis, endoplasmic reticulum stress, unfolded protein response

## Abstract

Chronic stress leads to immunosuppression and induces splenocyte apoptosis. STAT3 is a transcription factor that regulates immunity and apoptosis; however, it is unclear whether the increased expression of phosphorylated STAT3 (p-STAT3) observed in chronic stress is related to splenocyte apoptosis. To explore the relationship between splenocyte apoptosis and STAT3 in chronic stress, we treated rats undergoing a 21-day chronic restraint stress program with the STAT3 inhibitor S3I-201. This chronic stress model was verified by observing rats’ behavior and measuring their serum corticosterone levels. Chronic stress led to increased expression of anti-inflammatory cytokines, and p-STAT3 inhibition enhanced splenocyte apoptosis in chronic stress. We detected key proteins in three apoptotic pathways to determine which pathway mediated increasing splenocyte apoptosis and found that the death receptor pathway was the main apoptotic pathway that occurred in the spleen during chronic stress. The unfolded protein response (UPR) was also activated, but the Bcl-2 family was not involved in chronic stress. P-STAT3 inhibition had no influence on the Bcl-2 family and the death receptor pathway; however, p-STAT3 inhibition disrupted the pro-survival function of the UPR by decreasing the expression of ATF6α and p-IRE1α. Furthermore, p-STAT3 inhibition activated endoplasmic reticulum stress by promoting the expression of CHOP, p-JNK, and procaspase-12. Collectively, these findings indicate that the increased p-STAT3 expression during chronic stress may promote splenocyte survival by activating the UPR. Consequently, STAT3 and the UPR may be considered as potential therapeutic targets for chronic stress in the future.

## Introduction

Stress is the response that occurs when the body is threatened by stressors (Johnson et al., 1992). It can be divided into acute stress and chronic stress depending on its duration. During chronic stress, the hypothalamic–pituitary–adrenal (HPA) axis is the main neuroendocrine system that mediates various physiological and pathological changes (Charmandari et al., 2005). However, prolonged activation of the HPA axis can inhibit the protective immune responses and/or aggravate pathological immune responses, which is one of the factors by which chronic stress influences the onset and progression of various clinical diseases (Schneiderman et al., 2005). It has been reported that chronic stress results in thymic degeneration, Th1/Th2 imbalance, splenocyte apoptosis, and so on (Dominguez-Gerpe and Rey-Mendez, 2001).

STAT3 is a critical transcription factor that participates in the repair of damaged tissues by promoting cell proliferation (Levy and Lee, 2002) and can also enhance the survival of cells by increasing the expression of anti-apoptotic proteins. Programmed cell death is mainly mediated by the death receptor, mitochondrial, and endoplasmic reticulum (ER) stress pathways. Multiple studies have shown that STAT3 promotes the expression of anti-apoptotic proteins of the Bcl-2 family, which play roles in the mitochondrial pathway to inhibit apoptosis (Haga et al., 2003; Zaanan et al., 2015; Maji et al., 2019). The overexpression of STAT3 reduces hepatocyte apoptosis mediated by Fas (Ivanov et al., 2001), and activation of STAT3 inhibits ER stress to reduce cardiomyocyte apoptosis (Wang et al., 2014). On the other hand, many studies have reported that STAT3 also inhibits cell proliferation, for instance, in mediating breast degeneration (Morris et al., 2020) and inhibiting proliferation in certain tumor types (Musteanu et al., 2010; Couto et al., 2012).

STAT3 is also widely involved in adaptive and innate immunity and is activated by multiple cytokines to either promote or inhibit inflammation (Kortylewski et al., 2005; Yu et al., 2009). Anti-inflammatory cytokines are released into the blood during chronic stress upon activation of the HPA axis. Chronic stress was shown to activate STAT3 via IL-10 in spleens of rats treated with a 3-day restraint process (Hutchins et al., 2013). STAT3 also plays an important part in the development and function of many immune cells. Mice lacking the STAT3 gene in macrophages and neutrophils have been reported to exhibit Th1 dominance (Takeda et al., 1999). Immunosuppression caused by chronic stress often involves a decrease in Th1 cytokine levels with a corresponding increase in Th2 cytokine levels (Elenkov and Chrousos, 1999). During chronic stress, phosphorylated STAT3 (p-STAT3) inhibition decreases the expression of IL-12, which mediates the differentiation of Th1 cells (Hutchins et al., 2013). Chronic stress also results in splenocyte apoptosis. However, whether and how STAT3 participates in splenocyte apoptosis in chronic stress remain unknown.

In this work, we investigated the relationship between the high expression levels of p-STAT3 and splenocyte apoptosis, using rats treated with a p-STAT3-specific inhibitor, S3I-201. Furthermore, we explored signaling pathways mediating splenocyte apoptosis in chronic stress, in particular, the effects of STAT3 inhibition in the spleen.

## Materials and Methods

### Animals

Adult male Wistar rats (180–220 g) were purchased from the Experimental Animal Center of Harbin Medical University (Harbin, China). All rats were bred under a 12 h/12 h light/dark cycle (lights on from 6:00 to 18:00) at a temperature of 22 ± 1° C and were given free access to water and food for 7 days. All animal studies were approved by the Animal Experimental Committee of Northeast Agricultural University, Harbin, China (IACUC: SRM-11). The experiments were carried out in accordance with the National Institutes of Health Guide for Care and Use of Laboratory Animals.

### Experimental Model

Thirty-six rats were randomized to six groups, with six rats in each group. The control (C) group consisted of untreated rats, the chronic stress (CS) group was treated with a restraint program, and the control + S3I-201 (C + S3I-201) group was treated with a p-STAT3 inhibitor every 2 days. In the chronic stress + S3I-201 (CS + S3I-201) group, rats were treated with a p-STAT3 inhibitor every 2 days while undergoing the 21-day restraint stress program, the control + DMSO (C + DMSO) group was treated with 5% dimethyl sulfoxide (DMSO) every 2 days, and rats in the chronic stress + DMSO (CS + DMSO) group were treated with 5% DMSO every 2 days while undergoing the 21-day restraint stress program.

Rats in the CS, CS + S3I-201, and CS + DMSO groups were restrained using fixators with good ventilation for 6 h daily (9:00–15:00) for 21 days.

The p-STAT3 inhibitor S3I-201 was dissolved in 100% DMSO (BioFroxx, Einhausen, Germany) and diluted with corn oil (Aladdin, Shanghai, China) to 5%. S3I-201 (5 mg/kg, MedChemExpress, NJ, United States) was administered intraperitoneally 1 h before the daily restraint program, every 2 days for 3 weeks (Johnson et al., 2013; Zhou et al., 2015; Huang et al., 2016).

During the 21-day restraint program, no food or water was available for any of the rats from 9:00 to 15:00; at other times, they could eat and drink freely.

### Open-Field Test

Rats were individually placed in the corner of a wooden square arena (100 cm × 100 cm × 40 cm) and allowed to explore freely for 3 min. On the monitor, the bottom of the box was divided into 25 squares (20 cm × 20 cm); the surrounding area was defined as the outer area, and the rest was the central area. The behavior of rats, including total distance of movement, line crossing number (frequency with which the rats crossed one of the grid lines with all four paws), center square duration (duration of time the mice spent in the central area), and number of rearing (frequency of both forelimbs being off the ground), was recorded using the SuperMaze software (Shanghai Xinruan Information Technology Co., Ltd, Shanghai, China) to verify that the model had been successfully established. The arena was cleaned with 70% ethanol and thoroughly dried between sessions.

### Measurement of Serum Corticosterone Levels

After the open-field test, all rats were sacrificed to collect blood and spleen samples for further experiments. Blood samples were stored at room temperature for 30 min. Blood serum was obtained by centrifugation at 3,000 rpm for 10 min at 4° C. The concentration of corticosterone in the blood serum was measured using a Rat Corticosterone ELISA Kit (Nanjing Jiancheng Bioengineering Institute, Nanjing, China) according to the manufacturer’s instructions.

### Histopathology

Spleen samples obtained from each group were immersed in 10% buffered formalin for 24 h. After gradient elution using ethanol and embedding in paraffin, spleens were sliced into 5-μm-thick sections and stained with hematoxylin and eosin (H&E) after routine dewaxing. Sections were observed under a light microscope (BX-FM; Olympus Corp, Tokyo, Japan), and images were captured using the camera (Canon, Tokyo, Japan) with the software provided.

### Detection of Apoptosis by TUNEL Assay

Paraffin-embedded spleen tissue sections were deparaffinized and rehydrated after warming for 30 min. Terminal transferase-mediated dUTP nick end labeling (TUNEL) of nuclei was performed using a TUNEL Apoptosis Assay Kit (11684817910, Roche, Basel, Switzerland) according to the manufacturer’s protocol. Images were acquired with a Nikon Eclipse Ni inverted microscope (TE2000, Nikon, Tokyo, Japan) after antifluorescence quenching. Five visual fields (magnification: 200×) of each section were randomly selected, and the number of positive nuclei in spleens was counted using Image-Pro Plus 6.0 software (Media Cybernetics, MD, United States). Apoptosis was assessed using the following formula: apoptosis index = number of positive nuclei/number of all nuclei × 100%.

### Western Blotting

Small sections of the spleens were lysed in RIPA lysis buffer (P0013B, Beyotime Biotechnology, Shanghai, China) supplemented with a Roche protease inhibitor tablet (4906837001, Roche, Basel, Switzerland) and phenylmethyl sulfonyl fluoride (ST506, Beyotime Biotechnology). After homogenization with a Tissue Prep instrument, spleen lysates were centrifuged at 12,000 rpm for 10 min at 4° C, and the supernatant was collected. Protein quantification was performed using an Enhanced BCA Protein Assay Kit (P0012, Beyotime Biotechnology). Lysates were denatured using loading buffer and heating at 100° C for 10 min. Protein samples were separated using sodium dodecyl sulfate polyacrylamide gel electrophoresis and then transferred to polyvinylidene fluoride membranes (Millipore Sigma, Merck KGaA, Darmstadt, Germany). After blocking in 5% non-fat milk for 2 h at room temperature, the membranes were incubated in primary antibody dilution buffer overnight at 4° C. After washing in TBST (Tris-buffered saline and 0.1% Tween 20), membranes were incubated with horseradish peroxidase-conjugated secondary antibodies (ZDR-5306 and ZDR-5307, 1:10,000, ZSGB-BIO, Beijing, China) for 2 h at room temperature. Meilunbio fg super sensitive ECL luminescence reagent (MA0186, Dalian Meilun Biotechnology Co., Ltd, Dalian, China) was used as a chemiluminescence substrate. Immune-reactive protein bands were captured with a Tanon 5200 imaging system (Biotanon, Shanghai, China). The immunoblots were quantified and analyzed using ImageJ software. Antibodies to Phospho-STAT3^Tyr705^ (#9145, 1:1,000), STAT3 (#9139, 1:1,000), cleaved caspase-3 (#9664, 1:1,000), caspase-3 (#9662, 1:1,000), and phospho-eIF2α^Ser51^ (#3398, 1:1,000) were obtained from Cell Signal Technology (Danvers, USA). Antibodies to caspase-8 (sc-81656, 1:500), CHOP (sc-166682, 1:500), caspase-12 (sc-21747, 1:500), phospho-JNK^Thr183&*Tyr*185^ (sc-6254, 1:500), JNK (sc-7345, 1:500), and ATF6α (sc-166659, 1:750) were obtained from Santa Cruz (Dallas, USA). Phospho-IRE1α^S724^ (AP0878, 1:2,000) was obtained from Abclonal Technology (Wuhan, China). Antibodies to IL-10 (WL03088, 1:500), IL-6 (WL02841, 1:1,000), Bax (WL01637, 1:1,000), Bcl-2 (WL01556, 1:1,000), and Bcl-xL (WL03353, 1:1,000) were obtained from Wanleibio (Shenyang, China). The antibody to β-actin (TA-09, 1:10,000) was obtained from ZSGB-BIO (Beijing, China).

### Statistical Analysis

All data were analyzed using PASW Statistics 18 software (SPASS, IL, United States) and expressed as mean ± standard error of the mean (SEM). Poisson regression was performed when the dependent variable was the count variable (line crossing number and rearing number). Unpaired Student’s *t*-test was used to compare two sets of data. One-way analysis of variance (ANOVA) testing was performed with Tukey’s *post hoc* analysis to compare multiple sets of data. Graphs were generated using GraphPad Prism 5 (GraphPad Software for Windows Inc., San Diego, CA, United States). In all analyses, *p* < 0.05 was considered to indicate statistical significance and *p* < 0.01 to indicate extremely significant results.

## Results

### Chronic Restraint Stress Induces Anxiety-Like Behaviors and Affects HPA Axis Activity

**FIGURE 1:**
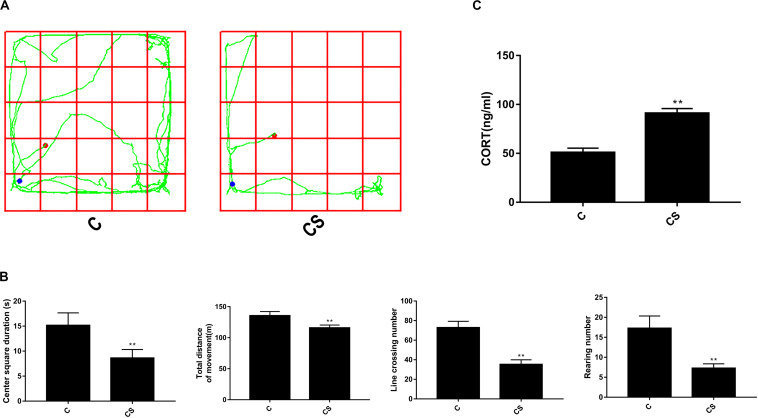
Chronic stress significantly changes open-field test results and corticosterone levels in rat serum. **(A)** The rats’ motion trails of the C and CS groups in the open-field test. **(B)** After 21 days of restraint stress, the center square duration, the total distance of movement, the rearing number, and the line crossing number were all significantly decreased compared with the C group. **(C)** Serum corticosterone levels increased significantly after 21 days of chronic stress compared with the C group. Data are presented as the mean ± SEM. *p < 0.05, **p < 0.01 compared with the C group, *n* = 6, Poisson regression or Student’s *t* test.

To verify successful establishment of our chronic stress model, we tested the behavior of rats using an open-field test after 21 days of restraint. Figure 1A shows motion trails of rats in the C and CS groups. As shown in Figure 1B, rats in the C group demonstrated excitement-like behaviors in the open-field test. Compared with those in the C group, stressed rats showed decreased center square duration and lower total distance of movement. The rearing number and line crossing number were also significantly reduced after exposure to chronic stress.

To examine whether chronic restraint stress affected HPA axis activity, we measured corticosterone levels in blood serum samples. As shown in Figure 1C, the level of corticosterone in the CS group increased significantly compared with that of the C group (90.94 ± 4.90 and 50.75 ± 4.59 ng/ml, respectively; *p* < 0.01). These results demonstrate that 21 days of restraint stress successfully induced biochemical and behavioral features consistent with chronic stress.

### IL-10 Mediates STAT3 Phosphorylation in Chronic Restraint Stress

**FIGURE 2:**
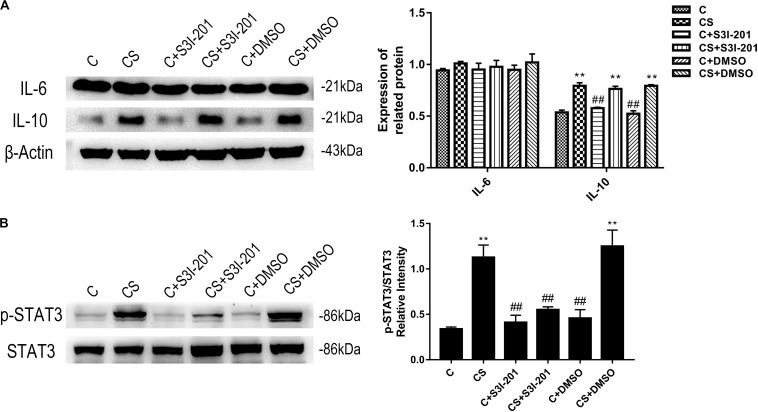
Chronic stress increases the expression levels of IL-10 and p-STAT3. The expression level of IL-6 was not affected by chronic stress. In addition to reducing p-STAT3 levels, p-STAT3 inhibition did not affect IL-10 and IL-6 expression levels. **(A)** Western blot showing the relative protein levels of IL-10 and IL-6. **(B)** Western blot showing the relative protein levels of p-STAT3/STAT3. Data are presented as the mean ± SEM. *p < 0.05, **p < 0.01 compared with the C group; ^#^*p* < 0.05, ^##^*p* < 0.01 compared with the CS group, *n* = 6, one-way ANOVA with Tukey’s *post hoc* test.

The HPA axis influences immunologic function through glucocorticoid secretion. Our study measured the expression of IL-10, an anti-inflammatory cytokine, and IL-6, an inflammatory cytokine; the function of both cytokines requires STAT3 activation. As shown in Figure 2, chronic stress promoted splenic IL-10 expression, which was not affected by p-STAT3 inhibition or vehicle treatment. IL-6 expression remained stable in response to chronic stress, p-STAT3 inhibition, and vehicle treatment. We also examined the expression of p-STAT3 in the spleen and found that chronic stress significantly increased p-STAT3 expression compared with that in the C group. Treatment with S3I-201 before initiation of restraint stress significantly attenuated the increase in p-STAT3 expression in response to chronic stress.

### P-STAT3 Inhibition Promotes Spleen Damage Caused by Chronic Stress

**FIGURE 3:**
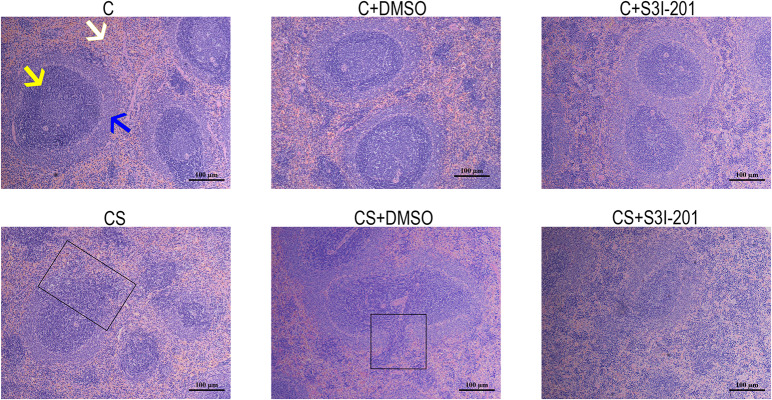
Effect of p-STAT3 inhibition on chronic stress induces spleen damage. Rat spleen tissue sections were stained with H&E (magnification: 100×). The yellow arrow indicates the red pulp, the white arrow indicates the white pulp, and the blue arrow indicates the marginal zone. The black box indicates the disappearance of the marginal zone.

H&E staining was used to evaluate histopathological alterations in the spleen in response to chronic stress. As shown in Figure 3, spleens in the C group showed typical histological architecture characterized by round or oval white pulp and red pulp, with a prominent marginal zone located between them, in the C group. White pulp, the main site of immunological function in the spleen, is mostly composed of lymphocytes and macrophages, whereas red pulp is made up of venous sinuses and a large variety of cell types. In response to chronic stress, spleens showed a reduction in white pulp and partial disappearance of the spleen marginal zone. Spleens of rats in the CS + S3I-201 group had more severe atrophy of white pulp and more loss of the marginal zone compared with those in the CS group. No changes to spleen histological structure were observed in normal rats treated with 5% DMSO or S3I-201.

### P-STAT3 Inhibition Promotes Splenocyte Apoptosis

**FIGURE 4:**
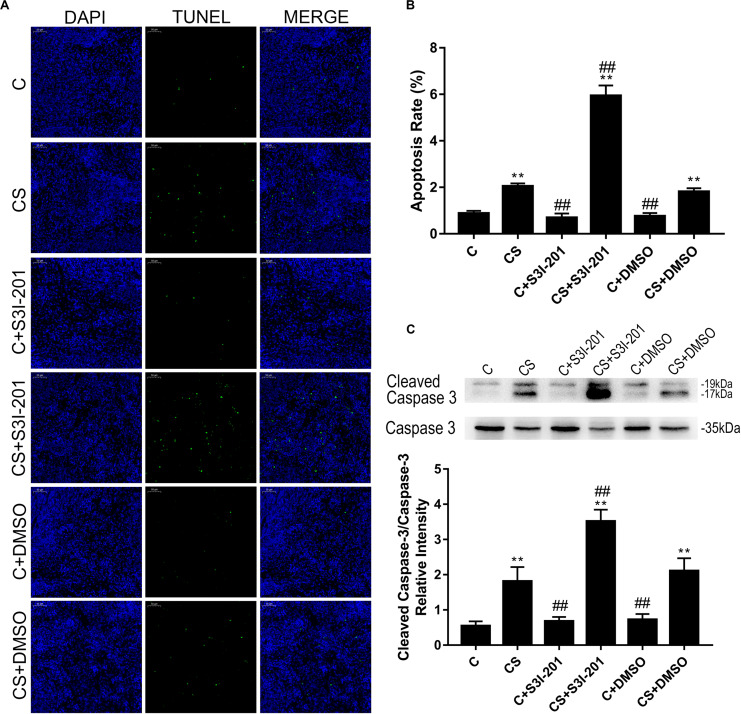
p-STAT3 inhibition enhances splenocyte apoptosis induced by chronic stress. **(A)** TUNEL assay on the splenic tissue of rats (magnification: 200×). TUNEL-positive splenocytes are stained green, and DAPI-stained nuclei are blue. **(B)** Quantitative results of TUNEL analysis. **(C)** Western blot showing relative protein levels of cleaved caspase-3/caspase-3. Data are presented as the mean ± SEM. *p < 0.05, **p < 0.01 compared with the C group; ^#^*p* < 0.05, ^##^*p* < 0.01 compared with the CS group, *n* = 6, one-way ANOVA with Tukey’s *post hoc* test.

We confirmed apoptosis in spleens by TUNEL assay and western blotting. As shown in Figures 4A,B, TUNEL-positive staining of nuclei was observed in splenic tissues of rats exposed to chronic stress. However, the splenic tissues of rats treated with S3I-201 before initiation of stress showed more TUNEL-positive nuclei staining compared with those of the untreated or vehicle-treated chronic stress group.

Furthermore, the ratio of cleaved caspase-3/caspase-3 (Figure 4C) supported the finding that p-STAT3 inhibition aggravated splenocyte apoptosis in response to chronic stress. Neither vehicle nor S3I-201 treatment induced splenic apoptosis in normal rats.

### P-STAT3 Inhibition Activates the ER Stress Pathway in Chronic Stress

**FIGURE 5:**
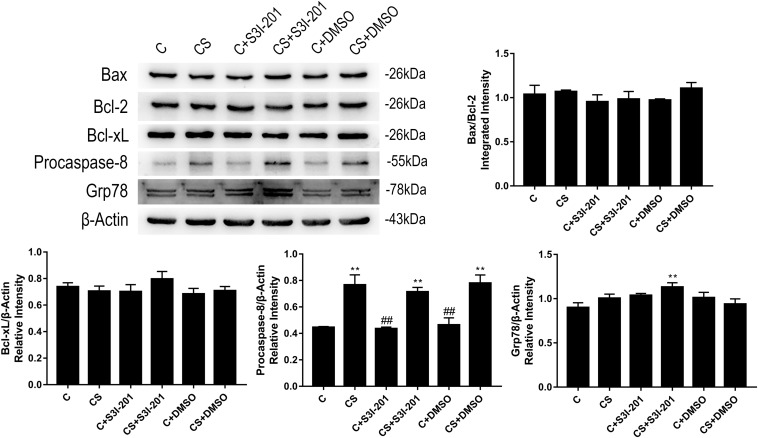
p-STAT3 inhibition activated the ER stress pathway instead of the mitochondrial apoptotic pathway or death receptor pathway. Western blot showing the relative protein levels of BAX/Bcl-2, Bcl-xL, procaspase-8, and Grp78. Data are presented as the mean ± SEM. *p < 0.05, **p < 0.01 compared with the C group; ^#^*p* < 0.05, ^##^*p* < 0.01 compared with the CS group, *n* = 6, one-way ANOVA with Tukey’s *post hoc* test.

To investigate the mechanism of enhanced splenic apoptosis following p-STAT3 inhibition in chronic stress, we measured the expression of key proteins in the three classical apoptotic pathways: the mitochondrial apoptotic pathway, the death receptor pathway, and the ER stress pathway. As shown in Figure 5, Bcl-2, Bax, and Bcl-xL expression did not significantly change in response to chronic stress or p-STAT3 inhibition. The expression of procaspase-8 was significantly increased in the CS group compared with the C group. However, procaspase-8 expression in the CS + S3I-201 group was similar to that of the CS group. The expression of Grp78, which dissociates from three ER transmembrane receptors to trigger ER stress, was higher in the CS + S3I-201 group than in the C group.

**FIGURE 6:**
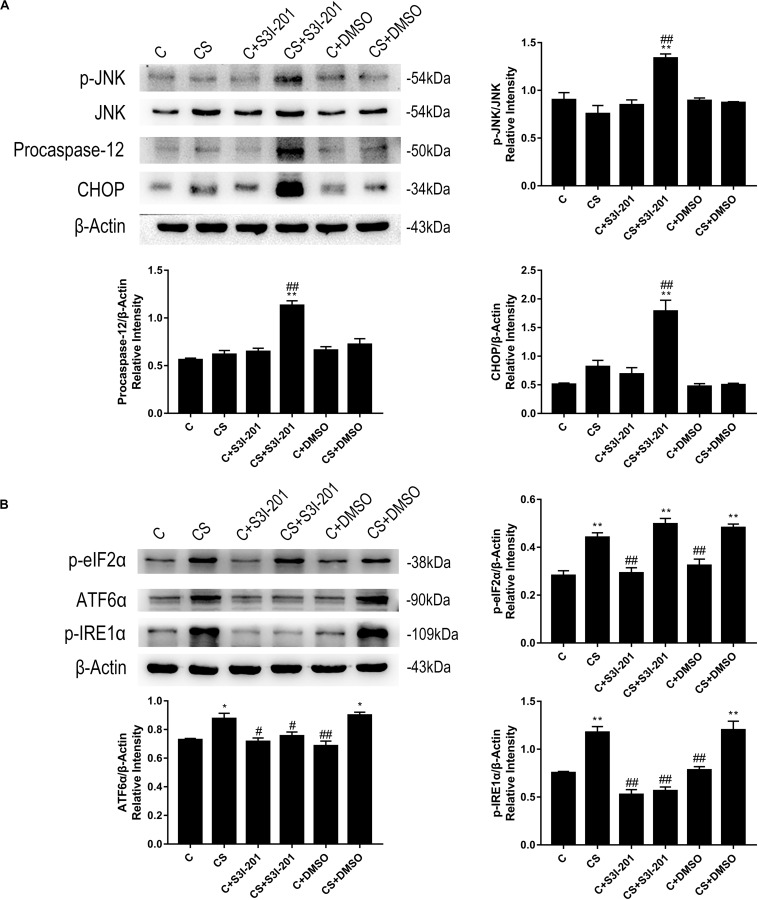
p-STAT3 inhibition activated ER stress by inhibiting the expressions of ATF-6α and p-IRE1α to disturb UPR. **(A)** Western blot showing the relative protein levels of p-JNK/JNK, procaspase-12, and CHOP. **(B)** Western blot showing the relative protein levels of p-eIF2α, ATF-6α, and p-IRE1α. Data are presented as the mean ± SEM. *p < 0.05, **p < 0.01 compared with the C group; ^#^*p* < 0.05, ^##^*p* < 0.01 compared with the CS group, *n* = 6, one-way ANOVA with Tukey’s *post hoc* test.

Based on these findings, we examined the expression of key proteins in ER stress-mediated apoptosis. As shown in Figure 6A, rats in the CS + S3I-201 group exhibited significantly increased expression levels of p-JNK, procaspase-12, and CHOP compared with those in the other five groups. Chronic stress alone did not affect the expression of these proteins. To further investigate the effects of chronic stress and p-STAT3 inhibition on ER stress, we assessed the expression of key proteins in each of the three distinct branches of the ER stress pathway (Figure 6B). Notably, the expression levels of p-eIF2α, ATF6α, and p-IRE1α were increased in the CS group, but the chronic stress-induced increased expression levels of ATF6α and p-IRE1α were attenuated in the CS + S3I-201 group.

## Discussion

Physical or psychological stressors trigger pressure-sensitive baroreceptor signaling that can activate the HPA axis (Johnson et al., 1992). Activation of HPA axis signaling, which has profound effects on the brain, is involved in the regulation of behavior and emotions (Naert et al., 2011). Here, we validated the successful establishment of a chronic restraint stress model by demonstrating changes in serum hormone levels and rat behavior. The open-field test is a common method to evaluate autonomous behavior, inquiry behavior, and tension among experimental animals in new environments. Although the total distance of movement and rearing number are typically used as measures of locomotor activity, they are also measures of exploration. Higher distance and rearing frequency measurements indicate more locomotion and exploration. Center square duration and line crossing number are measures of exploratory behavior. Accordingly, high center square duration and crossing frequency indicate high levels of exploratory behavior. The rats in the CS group showed less locomotor activity and lower levels of exploratory behavior in the open-field test, consistent with anxiety-like behaviors induced by chronic restraint stress.

Prolonged activation of the HPA axis not only affects the behavior of rats but also influences their immune homeostasis. High glucocorticoid levels promote the secretion of anti-inflammatory cytokines while inhibiting the secretion of pro-inflammatory cytokines. The elevated expression of IL-10, a classical anti-inflammatory cytokine, is caused by chronic stress and is considered a biomarker of immunosuppression. STAT3 can be activated by a variety of cytokines. However, blockade of IL-10 receptor in chronic stress decreases the expression of p-STAT3 (Hu et al., 2014), suggesting that the elevation expression of p-STAT3 in chronic stress is mediated, at least partially, by IL-10.

The spleen has an integral role in cellular and humoral immunity in adult rats. Damage to the spleen caused by chronic stress was observed by H&E staining in our study. Increased splenic damage was accompanied with the elevated expression of p-STAT3. The p-STAT3 inhibitor S3I-201 was administered to the rats to determine whether elevated p-STAT3 expression mediated splenic damage. Unexpectedly, S3I-201 enhanced splenic damage due to chronic stress but caused no spleen abnormality in unstressed rats. TUNEL assays and western blotting results also demonstrated that chronic stress caused splenocyte apoptosis, which was enhanced by p-STAT3 inhibition. Similarly, p-STAT3 inhibition in unstressed rats did not influence apoptosis. Our findings may be explained by the role of STAT3 in inhibiting apoptosis. STAT3 has been shown to be overactivated in a variety of tumors (Epling-Burnette et al., 2001; Siddiquee et al., 2007; Bollrath et al., 2009; Chen and Zhang, 2017), including solid tumors and hematological malignancies. Persistent STAT3 signaling leads to uncontrolled nuclear gene expression, which results in the growth and survival of tumor cells (Yu and Jove, 2004). *STAT3* gene knockdown or p-STAT3 inhibition activates the expression of pro-apoptosis proteins to mediate apoptosis of cancer cells (Guha et al., 2019). Activation of STAT3 in normal cells can also result in the activation of anti-apoptotic signals, which potentially indicates a mechanism by which chronic psychological stress promotes tumorigenesis (Feng et al., 2012). Our results demonstrate that S3I-201 injection did not cause damage to the spleen; however, STAT3 deficiency resulted in embryonic lethality (Takeda et al., 1997), possibly owing to the low expression of p-STAT3 in adult rats.

We examined key proteins in each of the three apoptotic pathways to identify the pathway that mediates enhanced apoptosis in response to S3I-201 treatment in chronic stress. Members of the Bcl-2 family are important regulators of the mitochondrial apoptotic pathway that sense abnormalities in cellular or mitochondrial function and terminate the injury process or initiate apoptosis accordingly (Murphy et al., 2005). Western blotting results indicated that Bcl-2, Bax, and Bcl-xL expressions were not influenced by chronic stress, suggesting that splenocyte apoptosis in response to chronic stress is not mediated by the Bcl-2 family. Furthermore, our findings indicate that the enhanced apoptosis in response to p-STAT3 inhibition in chronic stress is not mediated by the Bcl-2 family either. Although many studies have demonstrated that the Bcl-2 family is regulated by STAT3 (Niss et al., 2015; Liu et al., 2017), our results suggest that the Bcl-2 family members were not targets for STAT3 under the present conditions.

The formation of a death-inducing signaling complex (DISC), consisting of procaspase-8, Fas-associated with death domain protein (FADD), and tumor necrosis factor receptor (TNFR), is required for apoptosis mediated by the death receptor pathway (Tummers and Green, 2017). After formation of the DISC, procaspase-8 is auto-activated through autoproteolytic cleavage to form caspase-8, which triggers the execution phase of apoptosis (Elmore, 2007). Our western blotting results indicated that chronic stress led to an increased procaspase-8 expression, which was not influenced by p-STAT3 inhibition. Therefore, splenocyte apoptosis in our chronic stress model was mediated by the death receptor pathway, consistent with the finding of Yin et al. (2000), who reported CD95-mediated splenocyte apoptosis in response to chronic stress. The data suggest that STAT3 did not influence the death receptor pathway directly in our model, although it has been previously reported that STAT3 overexpression significantly inhibits caspase-8 activation to reduce liver damage (Haga et al., 2003). STAT3 is also known to cooperate with c-JUN to inhibit Fas transcription (Ivanov et al., 2001). Whether the overexpression of STAT3 in chronic stress influences the death receptor pathway remains to be determined.

ER stress is a relatively newly discovered pathway that has been shown to mediate apoptosis (Yang et al., 2015). Under certain physiological and pathophysiological conditions, the accumulation and aggregation of unfolded proteins affect the normal physiological function of the ER, which results in ER stress (Novosyadlyy et al., 2008). The unfolded protein response (UPR), which is mediated by three ER transmembrane receptors (PERK, ATF6, and IRE1), is a pro-survival response to reinstate normal ER function against the deleterious effects of ER stress. If the accumulation and aggregation of unfolded protein persist, signaling switches from pro-survival to pro-apoptotic (Liu et al., 2015). Increased expression levels of CHOP, procaspase-12, and JNK are indicative of apoptosis mediated by ER stress. Our western blotting results indicate that chronic stress does not promote the expression of those three proteins. However, p-STAT3 inhibition during chronic stress activated ER stress-mediated apoptosis by enhancing the expression levels of CHOP, procaspase-12, and p-JNK.

Following this finding, we measured the expression levels of p-eIF2α, ATF6α, and p-IRE1α. In the UPR, eIF2α slows down protein translation via phosphorylation to prevent oxidative stress and apoptosis (Tabas and Ron, 2011). ATF-6α is the key protein that regulates ER quality control (Yamamoto et al., 2007). IRE1α, which exists in all cells, is the last branch to be activated during the UPR in the decision of whether to initiate pro-survival or pro-apoptotic downstream signaling (Tabas and Ron, 2011). These three proteins each represent one branch in the UPR. Chronic stress significantly enhanced the expression of these three proteins, in contrast to CHOP, procaspase-12, and p-JNK, which were unaffected. A similar finding was observed in tumor-associated macrophages, in which IL-4 synergized with IL-10 to activate the UPR via STAT3 (Yan et al., 2016). Remarkably, p-STAT3 inhibition did not influence the expression of p-eIF2α, whereas both ATF6α and p-IRE1α showed the reduced expression in response to p-STAT3 inhibition. In our view, these results suggest that STAT3 regulates the latter two branches of UPR signaling to disrupt the pro-survival function of the UPR.

Notably, in the C and CS groups, upstream and downstream proteins of the UPR showed the totally different expression tendencies. We hypothesize that p-STAT3 inhibition blocks the UPR-mediated compensatory cell survival mechanism. During chronic stress, IRE1α and ATF6 cooperate with PERK in pro-survival roles to degrade improperly folded proteins and increase the folding capacity of the ER. After treatment with S3I-201, generation of ATF6α and p-IRE1α may be suppressed, which disrupts the pro-survival function of the UPR and leads to ER stress-mediated apoptosis. Yoshikawa et al. (2015) demonstrated that *ATF6*α-deficient mouse brains exhibited higher rates of neuronal death after cerebral ischemia. Zhang et al. (2015) reported that IRE1α deficiency resulted in the increased expression of CHOP, which induced apoptosis in intestinal epithelial cells. Although it has been demonstrated that ATF6α (Yoshikawa et al., 2015) and IRE1α (Kimura et al., 2012; Liu et al., 2015) influence the expression of STAT3, it remains unknown how STAT3 influences the expression of ATF6α and IRE1α. No STAT3-specific agonists are available for administration *in vivo*; therefore, we were unable to overexpress p-STAT3 to verify whether the UPR was enhanced and apoptosis was attenuated. The next step will be to determine the relationship between STAT3 and the UPR in an *in vitro* model of chronic stress.

Our results elucidate in part the role of STAT3 and the activation of the UPR in chronic stress. These data also confirm the relationship between STAT3 and ER stress. To our knowledge, this is the first report of the activation of the UPR and the relationship between STAT3 and ER stress in chronic stress. Our preclinical study provides new evidence regarding the function of STAT3 in chronic stress and identifies potential new therapeutic targets for the development of anti-stress drugs.

## Conclusion

Our results indicate that splenocyte apoptosis in response to chronic stress is mediated by the death receptor pathway. In particular, the spleen activates the UPR in response to chronic stress, and p-STAT3 inhibition enhances splenocyte apoptosis by inhibiting the UPR and activating ER stress.

## Data Availability Statement

The datasets generated for this study are available on request to the corresponding author.

## Ethics Statement

The animal study was reviewed and approved by Animal Experimental Committee of Northeast Agricultural University.

## Author Contributions

MS and HF helped to design the study, analyzed the data, and wrote the manuscript. HY, YC, and BL analyzed the data and drafted the manuscript. MS, CW, and XF performed the experiments and analyzed the data. All authors read and approved the final manuscript.

## Conflict of Interest

The authors declare that the research was conducted in the absence of any commercial or financial relationships that could be construed as a potential conflict of interest.
